# The prion 2018 round tables (I): the structure of PrP^Sc^

**DOI:** 10.1080/19336896.2019.1569450

**Published:** 2019-01-15

**Authors:** Ilia V. Baskakov, Byron Caughey, Jesús R. Requena, Alejandro M. Sevillano, Witold K. Surewicz, Holger Wille

**Affiliations:** a Center for Biomedical Engineering and Technology and Department of Anatomy and Neurobiology, University of Maryland School of Medicine, Baltimore, MD, USA; b Laboratory of Persistent Viral Diseases, Rocky Mountain Laboratories, National Institute of Allergy and Infectious Diseases, National Institutes of Health, Hamilton, MT, USA; c CIMUS Biomedical Research Institute and Department of Medical Sciences, University of Santiago de Compostela-IDIS, Santiago de Compostela, Spain; d Departments of Pathology and Medicine, University of California San Diego, La Jolla, CA, USA; e Departments of Pathology, and of Physiology and Biophysics, Case Western Reserve University, Cleveland, OH, USA; f Department of Biochemistry and Centre for Prions and Protein Folding Diseases, University of Alberta, Edmonton, AB, Canada

**Keywords:** PrP^Sc^ structure, PrP^Sc^ syalilation, 4-rung β-solenoid, PIRIBS, PrP23-144 infectious amyloid, cryo-electron microscopy, solid state NMR, Prion2018

## Abstract

Understanding the structure of PrP^Sc^ is without doubt a *sine qua non* to understand not only PrP^Sc^ propagation, but also critical features of that process such as the strain phenomenon and transmission barriers. While elucidation of the PrP^Sc^ structure has been full of difficulties, we now have a large amount of structural information that allows us to begin to understand it. This commentary article summarizes a round table that took place within the Prion 2018 meeting held in Santiago de Compostela to discuss the state of the art in this matter. Two alternative models of PrP^Sc^ exist: the PIRIBS and the 4-rung β-solenoid models. Both of them have relevant features. The 4-rung β-solenoid model agrees with experimental constraints of brain derived PrP^Sc^ obtained from cryo-EM and X-ray fiber diffraction studies. Furthermore, it allows facile accommodation of the bulky glycans that decorate brain-derived PrP^Sc^. On the other hand, the infectious PrP23-144 amyloid exhibits a PIRIBS architecture. Perhaps, both types of structure co-exist.

## Introduction

‘It has not escaped our notice that the specific pairing we have postulated immediately suggests a possible copying mechanism for the genetic material’. With this famous understatement, Watson and Crick reflected, in their classic 1953 paper, upon the fact that contemplation of the structure of DNA, that they had just deciphered, was sufficient to understand the mechanism by which this molecule propagates [1]. PrP^Sc^ prions also propagate, although what propagates in that case is not a primary structure but rather the secondary, tertiary and quaternary structures of a specific conformation of PrP [2]. For DNA, pairing of the two helical chains within the double helix underpins the templating mechanism. For PrP^Sc^ prions, some specific elements within the PrP^Sc^ conformation must underpin the capacity of this structure to mold an incoming PrP molecule into a copy of itself. Thus, understanding the structure of PrP^Sc^ without doubt holds the key to an immediate understanding of PrP^Sc^ propagation. Furthermore, a number of critical features of the propagation process such as the strain phenomenon and transmission barriers will be immediately understood once the structure of PrP^Sc^ is known with sufficient detail.

Historically, the quest to elucidate the structure of PrP^Sc^ has been a difficult one, given that PrP^Sc^ is an analytical nightmare: difficult to isolate, insoluble in water, polymeric, and featuring variable amounts of post-translation modifications. Nevertheless, a large amount of structural information and constraints have been amassed over time by indefatigable researchers, allowing us to begin to understand the structure of this enigmatic molecule.

In this context, a round table took place within the Prion 2018 meeting held in Santiago de Compostela to discuss what we know about the structure of PrP^Sc^. Ilia Baskakov, Byron Caughey, Witold Surewicz, and Holger Wille presented their points of view, under the moderation of Alejandro M. Sevillano and Jesús R. Requena. Ample participation by the audience in the ensuing discussion ensured a wide representation of different opinions. Here is a summary of the discussion.

## Main

### 1. Prion diversity

In addition to the generally intractable biophysical properties of PrP^Sc^ noted above, a number of other issues complicate PrP^Sc^ structure determination. First is the multitude of prion strains as well as disease-associated, but not transmissible, aggregates of PrP that must be explained. Many lines of evidence indicate that the PrP^Sc^ of different prion strains has different underlying self-propagating structures. Even for a given prion strain, wide ranges of sizes, ultrastructures, and biochemical characteristics have been observed, and some of these characteristics can be profoundly affected by the type of host animal, e.g. whether they expressed GPI-anchored or anchorless PrP^C^ [3]. Prion infectivity has been associated with particles ranging from small non-fibrillar PrP oligomers to amyloid fibrils hundreds of nm in length [4]. This raises the question of whether this range represents a size continuum of particles with essentially the same core structure, or more fundamentally distinct arrangements of monomers such as those indicated by PrP^Sc^ preparations with both amyloid fibrils and non-amyloid 2-D crystalline arrays (see below). Synthetic recombinant PrP prions or prion-like fibrillar assemblies have been described that can all propagate indefinitely *in vitro* but, when inoculated into animals, can range from being biologically inert to fully infectious, pathogenic, and transmissible in subsequent passages [e.g. 5–13]. Recent evidence suggests that the difference between these biological effects can sometimes be correlated with conformational differences [14,15]. Collectively, the diversity of prions and prion-like PrP assemblies suggest that there may be no single PrP^Sc^ structure, and that diversity in conformation, and even basic multimer architecture, should be anticipated. Indeed, a number of fungal prions have been shown to have parallel in-register intermolecular β-sheet (PIRIBS) architectures [16], while another is a β-solenoid [17]. It is notable that the latter, the [Het-s] prion of *Podospora anserina*, is a functional, evolutionarily selected prion, whereas most other prions, including the PrP-based mammalian prions, are the accidental consequences of the refolding of proteins that have quite distinct conformations in their normal physiological states [16]. In any case, much remains to be resolved about PrP^Sc^ structure and the diversity thereof. Careful, unbiased and dispassionate evaluation of emerging data, and empirically-based models, should help us to solve the decades-long mystery of the various ways in which PrP can misfold, aggregate, and propagate to cause devastating neurodegenerative diseases.

### 2. The PIRIBS model

Although evidence suggests that many prions may have β-solenoid architectures (see below), there is also clear evidence that some PrP amyloids, both spontaneously nucleated [18,19] and prion-seeded [10] recombinant PrP amyloids can have PIRIBS architectures. These conclusions were discerned primarily from site-specific spin-labeling [18] and solid-state NMR experiments [10,19], which allowed determination of the basic architecture of the multimers without providing all-encompassing atomistic structures. *In silico* models of such structures are consistent with many empirical descriptors and constraints on PrP^Sc^ structures and appear to provide a plausible molecular basis for faithfully templated strain propagation [10]. However, as detailed in the following sections, PIRIBS models are not obviously consistent with evidence for 2D crystals, a 19.2 Å repeating unit along the fibril axis, or maximal glycosylation of all PrP monomers, as appears to be the case with some prion strains. Thus, although PIRIBS fibrils of PrP can easily be made and propagated *in vitro*, it remains to be determined whether such structures represent any of the diverse pathological self-propagating PrP aggregates of natural prion diseases.

### 3. The 4-rung β-solenoid model

The hypothesis that the structure of PrP^Sc^ is based on a β-helix or β-solenoid fold arose from studies of an alternative polymerization state for the N-terminally truncated PrP 27–30 [20]. Traditionally, PrP 27–30 polymerized into amyloid fibrils termed ‘prion rods’ [21], but here two-dimensional (2D) crystals formed by PrP 27–30 trimers were observed. By comparing the PrP 27–30 2D crystals with isomorphic 2D crystals from a redacted ‘mini-prion’ (PrP^Sc^106) [22,23], it became apparent that traditional (i.e. elongated) β-strand architectures could not accommodate the tight packing of protein molecules within the 2D crystals. Difference mapping between 2D crystals from these two prion forms allowed the creation of the first molecular model that included a β-helix architecture [20]. A further refinement of the electron microscopy-based analyzes and the modeling approach restricted the β-helix portion to a compact, four-rung β-helix fold [24]. In subsequent years, a variety of alternate models based on the β-helix or β-solenoid hypothesis were published (reviewed in 25).

The original β-helix models still retained some α-helices (the C-terminal helices B and C) from the native PrP^C^ structure [20,24], but a thorough re-evaluation of previously published spectroscopy data [25,26] and new H/D-exchange experiments argued against this assumption [27]. Nevertheless, the data re-evaluation and the new experiments were still compatible with the β-helix/β-solenoid hypothesis, but reduced the number of restraints as the previously α-helical part of the protein became now available to adopt β-sheet structure as well.

Additional support for the β-helix hypothesis originated from X-ray fiber diffraction experiments, which revealed that the PrP^Sc^- and PrP 27–30-fold contains a four-rung β-solenoid architecture at its core [28]. The diffraction patterns included a series of reflections at 9.6, 6.4, and 4.8 Å, which corresponded to the second, third, and fourth order signals of a 19.2 Å repeating unit (i.e. 4 times 4.8 Å). Even the ‘mini-prion’ was found to include a four-rung β-solenoid core [29], which by itself is incompatible with any presumed, residual α-helices as the ‘mini-prion’ can only adopt a four-rung β-solenoid fold if those residues are included in the β-structure.

Next, limited proteolysis followed by mass spectrometry was used to locate loops and (partially) exposed residues in PrP 27–30 that could be cleaved by proteinase K [30]. The judicious use of denaturing conditions rendered the usually compact PrP 27–30-fold more amenable to this type of analysis. The resulting proteolytic fragments were compatible with the four-rung β-solenoid architecture, and a rough threading of the PrP primary structure onto this fold was developed [31].

Most recently cryo electron microscopy was employed to analyze the structure of PrP 27–30, and the results were found to be fully compatible with a four-rung β-solenoid architecture at the core of the infectious prion [32]. Here, a GPI-anchorless variant of PrP was used [3], which reduced the complexity of the resulting amyloid fibrils. Electron micrographs of individual amyloid fibrils were used to generate three-dimensional (3D) reconstructions, revealing the presence of two protofilaments. The protein density in these protofilaments could only be accommodated with the physical dimensions and an observed average molecular height of 17–19 Å, if a four-rung β-solenoid architecture was assumed [32]. Lastly, the β-solenoid hypothesis provides constraints for the replication of prions that involves templating on the first and/or last rung of the β-solenoid structure [33]. The steric constraints on the templating process that result as a consequence of the molecular architecture will be informative for future, higher-resolution analyzes of the structure of PrP^Sc^.

### 4. Constraints imposed by glycosylation

Several structural models of PrP^Sc^ that exhibit diverse PrP folding patterns including parallel in-register β-structures and two-, three- or four-rung β-solenoids have been proposed in recent years (reviewed in 33). In an effort to build a realistic molecular model of PrP^Sc^, our knowledge about PrP^Sc^ glycosylation should be taken into consideration. The first question to ask, is which of the proposed models can accommodate N-linked glycans? To address this question, a tri-antennary glycan with a size average of those found in PrP sialoglycoforms was used for modeling PrP^Sc^ [34]. In-register parallel β-sheet structure, the glycans of neighboring PrP molecules have to be spaced at a distance of 4.7 Å bringing them into substantial spatial overlap that precludes such arrangements [34]. Considerable spatial overlap between glycans still exists in two-rung solenoid that separates glycans at a distance of 2 × 4.7 Å. However, three- or four-rung solenoids permit recruitment of diglycosylated PrP molecules [34]. The result of modeling supports the hypothesis that glycans limit the diversity of folding patterns accessible to glycosylated PrP^C^.

To better understand how glycans might be enrolled in defining strain-specific PrP^Sc^ structures, two alternative views can be considered. According to one view, prion strains can partially overcome constraints imposed by glycans by selectively recruiting mono- and un-glycosylated PrP^C^ sialoglycoforms at the expense of diglycosylated sialoglycoforms. Only those PrP^C^ glycoforms are recruited that fit into strain-specific PrP^Sc^ structures. Alternative view proposes that recruitment is not selective, i.e. PrP^C^ sialoglycoforms are incorporated into PrP^Sc^ proportionally to their relative presentations in a pool of PrP^C^ molecules expressed in a cell. If this is the case, the spectrum of PrP^Sc^ structures is limited to those that can accommodate all sialoglycoforms. To answer the question on selectivity of recruitment, composition of PrP^Sc^ sialoglycoform was analyzed using 2D gels [35,36]. Because N-linked glycans carry negatively charged sialic acid residues, the sialoglycoforms can be separated in horizontal dimension of 2D according to their charge [37]. The 2D analysis revealed that PrP^Sc^ strains exhibits broad range of strain-specific selectivity with respect to PrP^C^ sialoglycoforms [34,35]. Consistent with the first mechanism, a group of strains shows strong preferences, as they excluded highly sialylated molecules as well as diglycosylated molecules [34,35]. At the same time, in support of the second mechanism, a group of strains did not display any preferences with respect to glycosylation or sialylation status [34,35]. Analysis across all examined strains revealed a great correlation between glycosylation and sialylation status of PrP sialoglycoforms within PrP^Sc^ [34,35]. This analysis also demonstrated a broad range of selectivity displayed by prion strains in recruiting PrP^C^ sialoglycoforms, ranging from non-selective to highly selective. Notably, for the group of non-selective strains, the composition of sialoglycoforms within PrP^Sc^ was very similar to that of PrP^C^.

**Figure 1.**
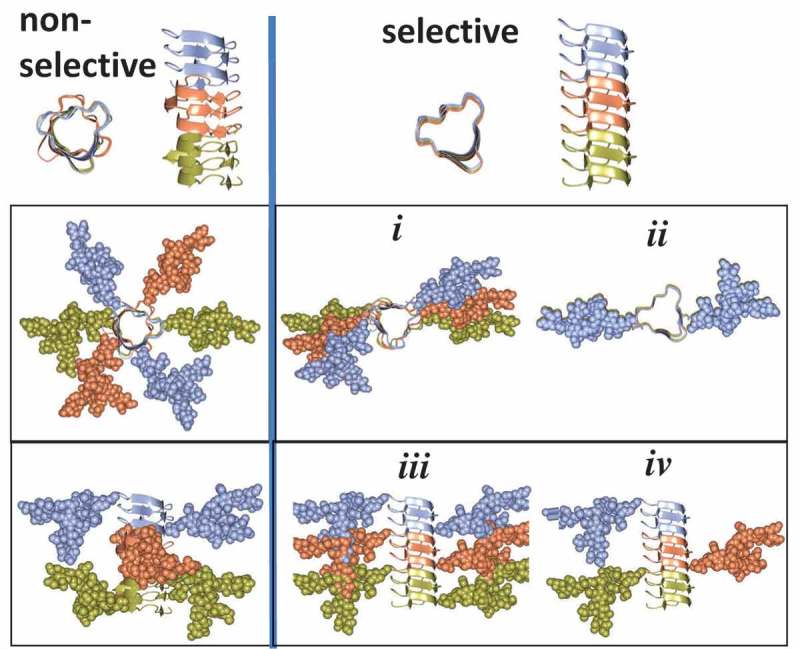
Schematic diagram illustrating differences in quaternary assembly between non-selective (left panels) and selective (right panels) strains. Non-selective strains can accommodate diglycosylated sialoglycoforms due to rotation between neighboring PrP molecules that allows spatial separation of glycans. In selective strains, the rotation between neighboring PrP molecules is very small (a) or absent (b). Recruitment of diglycosylated molecules by selective strains would lead to spatial interference between glycans (c). Negative selection of diglycosylated molecules helps to minimize spatial and electrostatic interference between glycans (d).

A broad range of selectivities displayed by prion strains could be attributed to strain-specific variations in quaternary structures and does not require significant variations in strain-specific folding patterns (Figure 1). In particular, quaternary assembly of non-selective strains could involve considerable twist or rotation between neighboring PrP molecules. Owing to such rotations, glycans of neighboring PrP molecules extend into different directions avoiding spatial interference and minimizing electrostatic repulsion between sialic acid residues (Figure 1). In strains that select against diglycosylated and highly sialylated PrP^C^, the rotation between neighboring PrP molecules is proposed to be very small or absent. Such modes of assembly can create spatial and electrostatic interference between glycans and limit recruitment of diglycosylated and highly sialylated PrP^C^ (Figure 1). The three- or four-rung solenoid models offer the best opportunity for accommodating both selective and non-selective strains.

To summarize our view on constraints imposed by glycosylation, prion strains display a broad range of selectivity toward PrP^C^ sialoglycoforms. Some strains recruit sialoglycoforms proportionally to their presentation in PrP^C^, whereas others avoid diglycosylated and highly sialylated PrP^C^ isoforms. Strain-specific ratio of the glycoforms within PrP^Sc^ is a result of negative selection of heavily sialylated PrP molecules with bulky glycans. The extent to which heavily sialylated glycoforms are excluded is believed to be controlled by a strain-specific structure. Glycan volume and electrostatic repulsion due to sialylation have to be taken into consideration for modeling PrP^Sc^ structures.

### 5. Lessons from studies with PrP23–144 amyloid fibrils

An important aspect of human prion diseases is the presence of a large number of mutations in the human PrP gene (*PRNP*) that segregate with familial CJD, Gerstmann-Sträussler-Scheinker (GSS) disease or fatal familial insomnia. All these disorders are autosomal dominant. One of these disease-related mutations, associated with GSS-like subtypes, is the tyrosine to stop codon mutation at position 145, resulting in a C-terminally truncated PrP fragment corresponding to residues 23–144 (PrP23-144) (38; 39). Bacterially expressed recombinant PrP23-144 from different species readily form amyloid fibrils under physiologically relevant conditions, and studies in vitro with this truncated PrP variant provided important insights into mechanistic principles of the conformational basis of species- and strain-dependent seeding specificity of prion protein amyloids [40,41].

The potential value of PrP23-144 amyloid as a model for exploring molecular aspects of mammalian prion propagation is further indicated by recent studies showing that mouse PrP23-144 fibrils are infectious, causing clinical prion disease in mice [42]. An intriguing and highly unusual feature of the latter disease is the accumulation in mouse brain of two types of self-propagating PK-resistant PrP fragments: one of them about 6–7 kDa in size (with N- and C-termini mapping to residues ~80–89 and 150–159, respectively) and the second one with molecular mass upon deglycosylation of ~17–18 kDa. While the shorter fragments are reminiscent of human PrPres in GSS subtypes, the longer fragments are similar to those observed in classical mouse-adapted scrapie strains.

The finding that PrP23-144 fibrils are infectious is of particular importance given that, in contrast to fibrils formed from full-length PrP, the former fibrils give rise to high quality solid-state NMR (ssNMR) spectra and, thus, are amenable to high-resolution structural characterization. Different types of ssNMR studies with human PrP23-144 amyloid fibrils published over the past ten years revealed that (i) The rigid β-core of the amyloid spans an approximately 30-amino acid segment that maps to residues ~112–140, with the reminder of the protein dynamically disordered; (ii) This rigid core region consists of three β-strands encompassing residues ~112–113 (strand 1), ~120–123 (strand 2), and ~130–140 (strand 3); and (iii) The core region displays a parallel in-register organization of β-strands [43–45]. Furthermore, a recently published structural model [46] reveals that these fibrils consist of two protofilaments with β-sheet regions running parallel to the long fibril axis. The compact hydrophobic core of constituent monomers consists of Ala, Gly and Val-rich segment between residues ~115–122, and this structure is stabilized by a highly specific interaction between the side chains of Ala117 in this hydrophobic core and Ile139 in the longest β-strand. Interestingly, a GSS-related substitution of Ala117 with Val disrupts this stabilizing interaction, resulting in a different amyloid fold. Apart from this high-resolution insight into the structure of human PrP23-144 amyloid, ssNMR studies also revealed the nature of structural differences between PrP23-144 amyloid fibrils from different species, providing a structural basis for understanding species-dependent seeding barriers [45].

Even though mouse PrP23-144 amyloid fibrils can seed in vivo the conversion of full-length PrP to an infectious, self-propagating structure that displays PK-resistance similar to that of classical scrapie strains, it is at present unknown whether the product of this seeding reaction retains the parallel in-register structural motif of the seed. In any case, structural and biological data for PrP23-144 amyloid clearly indicate that prion protein fibrils with parallel in-register organization can be infectious. Combined with recent evidence for a β-solenoid structure of anchorless prions, this raises an intriguing possibility that entirely different structural motifs may be present in distinct prion strains.

## Conclusions

The earlier large trove of structural models of PrP^Sc^ [47] has now shrunk to only two remaining models: the PIRIBS and the 4-rung β-solenoid models. Both of them have important features that make them relevant. The 4-rung β-solenoid model agrees with experimental constraints of brain derived PrP^Sc^ obtained through cryo-EM and X-ray fiber diffraction studies [28,32]. Furthermore, it allows facile accommodation of the bulky glycans present in brain-derived PrP^Sc^ [34]. However, the PrP23-144 amyloid, which exhibits a PIRIBS architecture [46] has also been demonstrated to be infectious [42]. How can these facts be reconciled? The most parsimonious explanation is that both types of structure co-exist. The first recombinant PrP prions were generated in 2004 using a technique that is known to yield amyloid fibers [5]. In retrospect, it is likely that these prions were structurally similar to amyloids produced at the later time, whose architecture is known to conform to the PIRIBS model, as assessed by site-directed spin labeling, solid-state NMR [10,18,19], and X-ray fiber diffraction analyzes [28]. In some cases, such PrP amyloids can propagate in the brain of recipient experimental animals without causing a clinical disease (v.g. 11,13). Atypical PrPres is one of the example of self-replicating and transmissible, yet clinically silent state [48–50]. However, upon second or third passage they evolve to classical PrP^Sc^ prions that lead to TSE disease [13,17,49–51]. This conformational transformation can be easily tracked biochemically, as the pattern of PK-resistant fragments evolves throughout passages. Therefore, it is conceivable that different types of PrP amyloids with a propagative, infectious properties can co-exist in the brain. Even though some of them exhibit a PIRIBS architecture, it is likely that the most infectious ones might exhibit an alternative 4-rung β-solenoid structure. Furthermore, PIRIBS structures might be able to template 4-rung β-solenoids and 4-rung β-solenoids might template PIRIBS amyloids, as was seen in the amyloid seeding assay [52]. The deformed templating model might provide a useful framework for explaining mutual templating of self-propagating structures with alternative folding patterns [53]. To add further layers of complexity, different subtypes of PIRIBS and 4-rung β-solenoid conformations/architectures might have different degrees of infectivity, and some of them may be even innocuous while maintaining the capacity to self-propagate [11,13,14,49]. However, it should be remembered that infectivity is an operational, not an absolute property of a potentially infectious agent. In this respect, thousands of British citizens are believed to harbor PrP^Sc^ in their bodies, but it will hopefully not cause any clinical neurodegenerative disease in their lifetime [54]. In this context, it might be necessary to acknowledge that PrP^Sc^ is not the only infectious PrP amyloid, that in fact some PrP^Sc^ strains and/or subtypes can propagate within brains without causing disease, just as some other propagative PrP amyloids that are not PrP^Sc^, and that interconversions among these could happen. In other words, that the terms ‘PrP^Sc^’ and ‘PrP prion’ might perhaps not be synonyms.
